# Prognostic value of wait time in nasopharyngeal carcinoma treated with intensity modulated radiotherapy: a propensitymatched analysis

**DOI:** 10.18632/oncotarget.7789

**Published:** 2016-02-29

**Authors:** Yu-Pei Chen, Yan-Ping Mao, Wen-Na Zhang, Lei Chen, Ling-Long Tang, Wen-Fei Li, Xu Liu, Guan-Qun Zhou, Rui Guo, Ying Sun, Tie-Bang Kang, Mu-Sheng Zeng, Jun Ma

**Affiliations:** ^1^ Sun Yat-sen University Cancer Center, State Key Laboratory of Oncology in South China, Collaborative Innovation Center for Cancer Medicine, Guangzhou, People's Republic of China

**Keywords:** nasopharyngeal carcinoma, wait time, intensity modulated radiotherapy, prognosis, propensity score matching

## Abstract

The aim of this study was to determine the prognostic value of wait time from histological diagnosis to primary treatmen for nasopharyngeal carcinoma (NPC) treated with intensity-modulated radiotherapy (IMRT). Between October 2009 and February 2012, a total of 1672 NPC patients were retrospectively analyzed. A cutoff value of > 4 weeks was used to define prolonged wait time. Matched patients according to the wait time were identified using propensity score matching (PSM), which was also used to identify matched patients for subsequent stratified analyses. Differences in progression-free survival (PFS), overall survival (OS), distant metastasis-free survival (DMFS), and locoregional relapse-free survival (LRFS) were estimated using the Kaplan–Meier method and Cox proportional hazards models. In total, 407 pairs of NPC patients were selected by PSM. The 3-year PFS rate was significantly lower for patients with a prolonged wait time (> 4 weeks) than for those with an acceptable wait time (*P* = 0.035). Stratified analyses revealed that the negative effects of a prolonged wait time occurred primarily in patients with advanced NPC without neoadjuvant chemotherapy (NACT; PFS:*P* = 0.040; DMFS:*P* = 0.028). In multivariate analysis, a prolonged wait time was found to be an independent unfavorable prognostic factor for PFS and DMFS in advanced-staged patients without NACT. These results suggest that a prolonged time (> 4 weeks) between diagnosis and primary radical radiotherapy is a disadvantage for NPC patients, particularly those with advanced disease receiving no NACT. Thus, it is necessary to optimize resources for decreasing this wait time, although additional studies are warranted to further clarify our findings.

## INTRODUCTION

Nasopharyngeal carcinoma (NPC) is a specific head and neck malignancy commonly observed in southern China, where the incidence is approximately 15–50 per 100,000 [1]. Radiotherapy (RT) is the mainstay treatment modality for nondisseminated NPC. With the advent of intensity modulated radiation therapy (IMRT) and combined chemotherapy-RT strategies, the management of NPC has been revolutionized. IMRT offers an improved target conformity and allows safer dose escalations, greatly improving locoregional control and gradually replacing two-dimensional conventional RT as the primary RT strategy for NPC [2]. Concurrent chemoradiotherapy (CCRT) with or without adjuvant chemotherapy (AC) has been demonstrated to be the most efficacious and is now recommended as a standard treatment for patients with locoregionally advanced NPC [3, 4]. A meta-analysis reported that additional neoadjuvant chemotherapy (NACT) may effectively decrease the distant metastasis rate and improve survival [5]. However, certain time-related factors such as the wait time from diagnosis to definitive RT, remain to be optimized for NPC [6].

For patients with neoplasms requiring radical surgery as the primary treatment, a prolonged time between diagnosis and primary treatment, i.e., the wait time, was found to be an important issue that reflected problems in the health care system, such as poor access to services, poor quality, and inefficiency [7]. A prolonged wait time can increase patient anxiety and negatively affect their prognosis [8, 9]. Previous studies found that a prolonged wait time is associated with shorter survival in patients with rectal cancer [9], breast cancer [10], and melanoma [11]. A meta-analysis found that a delay in the initiation of RT as the primary treatment was associated with an increase in the local recurrence rate for head and neck cancer [12], while Fortin et al. [13] found that a delay of > 40 days in radical RT decreased the survival of patients with early-stage head and neck cancer.

To the best of our knowledge, the prognostic value of the wait time has not been clearly demonstrated for NPC patients. In the era of two-dimensional conventional RT, Lee et al. [14] found that the wait time was not significantly associated with local failure in patients with T1 NPC. However, Chen et al. [6] recently reported some factors associated with a prolonged wait time, although their study lacked data regarding cancer stage and survival analyses.

From the abovementioned perspectives and according to the wide increase in the use of IMRT and chemotherapy-RT strategies, we conducted this study to investigate the prognostic value of the wait time for NPC patients with IMRT as the primary treatment. To balance the influence of covariates, we adopted the propensity score matching (PSM) method to compare survival outcomes and decrease potential bias [15].

## RESULTS

### Prognostic value of the wait time for NPC patients

From the original 1672 NPC patients, 407 pairs were selected by PSM (Table 1). The median wait time for patients with wait times of > 4 weeks and ≤ 4 weeks was 49 days (29–424 days) and 20 days (1–28 days), respectively. The median follow-up duration for the entire cohort was 38.42 months (1.27–58.80 months). Up to the last day of follow-up, a total of 23/814 (2.8%) patients died. Distant metastasis occurred in 47/814 (5.8%) patients, while locoregional recurrence occurred in 58/814 (7.1%) patients. The 3-year progression-free survival (PFS), overall survival (OS), distant metastasis-free survival (DMFS), and locoregional recurrence-free survival (LRFS) rates for the entire cohort were 88.8%, 97.5%, 94.3%, and 93.4%, respectively.

**Table 1:** Baseline characteristics of all 407 pairs of patients with nasopharyngeal carcinoma

Characteristic	Wait time ≤ 4 weeks (*n* = 407) No. (%)	Wait time > 4 weeks (*n* = 407) No. (%)	*P*^a^
Age			0.105
≤ 45	194 (47.7)	171 (42.0)	
> 45	213 (52.3)	236 (58.0)	
Sex			0.523
Male	305 (74.9)	297 (73.0)	
Female	102 (25.1)	110 (27.0)	
WHO pathology			0.624
Type I	3 (0.7)	1 (0.2)	
Type II/III	404 (99.3)	406 (99.8)	
T category			0.219
T1	111 (27.3)	107 (26.3)	
T2	74 (18.2)	94 (23.1)	
T3	210 (51.6)	189 (46.4)	
T4	12 (2.9)	17 (4.2)	
N category			0.493
N0	103 (25.3)	100 (24.6)	
N1	280 (69.8)	290 (71.3)	
N2	24 (5.9)	17 (4.2)	
N3	0 (0)	0 (0)	
Clinical stage			0.374
I	25 (27.3)	30 (7.4)	
II	145 (35.6)	158 (38.8)	
III	225 (55.3)	202 (49.6)	
IV	12 (2.9)	17 (4.2)	
Chemotherapy			0.534
No	82 (20.1)	75 (18.4)	
Yes	325 (79.9)	332 (81.6)	
ACE-27			0.264
≤ 1	389 (95.6)	395 (97.1)	
> 1	18 (4.4)	12 (2.9)	

Abbreviations: ACE-27 = Adult Comorbidity Evaluation-27; WHO = World Health Organization

a *P*-values were calculated using chi-square tests or Fisher's exact tests where indicated.

**Figure 1:**
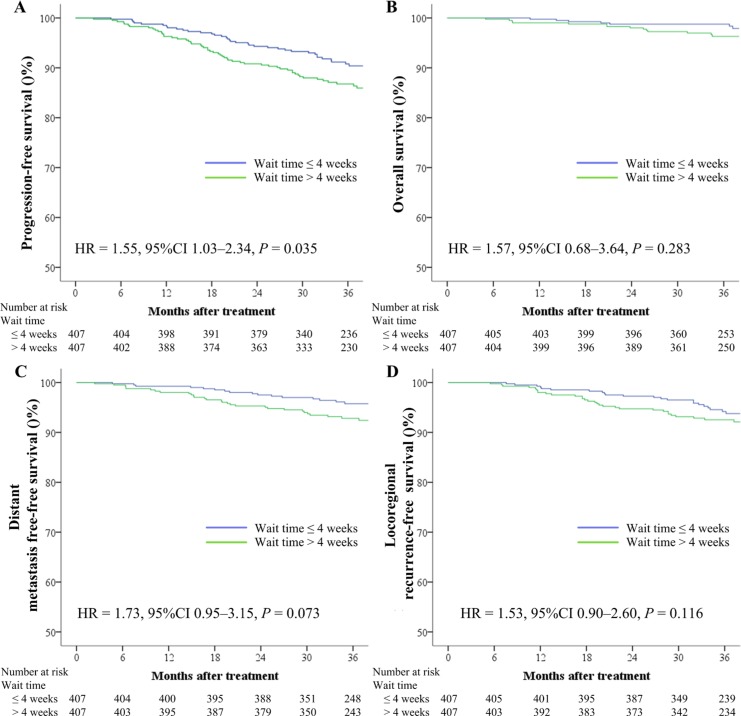
Kaplan–Meier survival curves based on the wait time (time between diagnosis and intensity-modulated radiotherapy) for progression-free survival (**A**), overall survival (**B**), distant metastasis-free survival (**C**), and locoregional recurrence-free survival (**D**) rates for 407 pairs of patients with nasopharyngeal carcinoma. CI = confidence interval, HR = hazard ratio.

The 3-year PFS rate was significantly lower for patients with a wait time of > 4 weeks (86.7%) than for those with a wait time of ≤ 4 weeks (90.8%; *P* = 0.035; Figure 1A). The difference in the 3-year DMFS rate (92.8% vs. 95.7%, respectively) between the two groups nearly reached statistical significance (*P* = 0.073; Figure 1C), while no significant differences were found for OS and LRFS (3-year OS: 96.3% vs. 98.8%, respectively, *P* = 0.283; 3-year LRFS: 92.5% vs. 94.2%, respectively, *P* = 0.116; Figure 1B, 1D). Multivariate analysis was performed to adjust for various prognostic factors, and consistent with the results of univariate analysis, it revealed that a wait time of > 4 weeks was an independent unfavorable prognostic factor for PFS [hazards ratio (HR), 1.55; 95% confidence interval (CI), 1.03–2.33; *P* = 0.037; Table 2].

**Table 2:** Multivariate analysis of prognostic factors for all 407 pairs of patients with nasopharyngeal carcinoma

Endpoint	Variable	HR (95% CI)	*P* ^a^
PFS	Wait time (> 4 vs. ≤ 4 weeks)	1.55 (1.03–2.33)	0.037
	N category (N1-3 vs. N0)	1.71 (1.00–2.92)	0.052
OS	Wait time (> 4 vs. ≤ 4 weeks)	NS	—
	T category (T3-4 vs. T1-2)	2.03 (0.83–4.92)	0.107
DMFS	Wait time (> 4 vs. ≤ 4 weeks)	1.77 (0.97–3.21)	0.063
	Gender (Male vs. female)	2.97 (1.17–7.52)	0.022
LRFS	Wait time (> 4 vs. ≤ 4 weeks)	1.53 (0.90–2.60)	0.116
	N category (N1-3 vs. N0)	1.83 (0.90–3.73)	0.096

Abbreviations: CI = confidence interval, DMFS = distant metastasis-free survival, HR = hazard ratio, LRFS = locoregional recurrence-free survival, NS = not significant, OS = overall survival, PFS = progression-free survival.

a *P*-values were calculated using an adjusted Cox proportional hazards model.

### Prognostic value of the wait time for NPC patients with and without NACT

Patients who received NACT generally exhibited a prolonged wait time. To further explore the prognostic value of the wait time for patients with and without NACT, we conducted a stratified analysis of 40 and 289 PSM-selected pairs of NPC patients with and without NACT, respectively (Supplementary Table S1).

**Figure 2:**
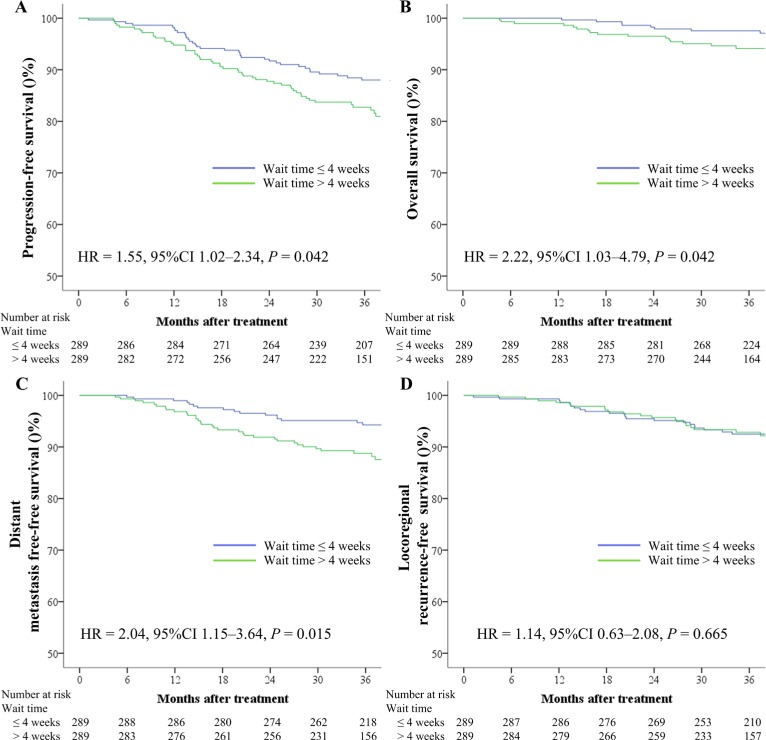
Kaplan–Meier survival curves based on the wait time (time between diagnosis and intensity-modulated radiotherapy) for progression-free survival (**A**), overall survival (**B**), distant metastasis-free survival (**C**), and locoregional recurrence-free survival (**D**) rates for 289 pairs of patients with nasopharyngeal carcinoma who did not receive neoadjuvant chemotherapy. CI = confidence interval, HR = hazard ratio.

With regard to patients who received NACT, the 3-year PFS (79.9% vs. 84.3%, respectively; *P* = 0.484), OS (95.0% vs. 92.4%, respectively; *P* = 0.600), DMFS (97.3% vs. 94.9%, respectively; *P* = 0.476), and LRFS (82.5% vs. 91.2%, respectively; *P* = 0.144) rates were comparable between patients with a wait time of > 4 weeks and those with a wait time of ≤ 4 weeks. However, with regard to patients who did not receive NACT, those with a wait time of > 4 weeks exhibited poorer 3-year PFS (82.7% vs. 88.0%, respectively; *P* = 0.042; Figure 2A), OS (94.1% vs. 97.5%, respectively; *P* = 0.042; Figure 2B), and DMFS (88.7% vs. 94.3%, respectively; *P* = 0.015; Figure 2C) rates compared to those with a wait time of ≤ 4 weeks. There was no significant difference in the 3-year LRFS rate between patients with a wait time of > 4 weeks and those with a wait time of ≤ 4 weeks (92.8% vs. 92.5%; *P* = 0.665; Figure 2D). Multivariate analysis showed that a wait time of > 4 weeks was an independent unfavorable prognostic factor for PFS, OS, and DMFS in NPC patients without NACT (Table 3).

**Table 3:** Multivariate analysis of prognostic factors for patients with nasopharyngeal carcinoma with and without NACT

Endpoint	Patients with NACT (*n* = 40 pairs)			Patients without NACT (*n* = 289 pairs)		
	Variable	HR (95% CI)	*P* ^a^	Variable	HR (95% CI)	*P* ^a^
PFS	Wait time (> 4 vs. ≤ 4 weeks)	NS	—	Wait time (> 4 vs. ≤ 4 weeks)	1.58 (1.03–2.41)	0.037
	Age (> 45 vs. ≤ 45)	3.95 (1.12–14.1)	0.033	T category (T3-4 vs. T1-2)	2.00 (1.24–3.22)	0.004
	—	—	—	N category (N1-3 vs. N0)	1.77 (0.91–3.46)	0.094
OS	Wait time (> 4 vs. ≤ 4 weeks)	NS	—	Wait time (> 4 vs. ≤ 4 weeks)	2.23 (1.03–4.82)	0.041
	—	—	—	T category (T3-4 vs. T1-2)	2.47 (1.06–5.79)	0.037
DMFS	Wait time (> 4 vs. ≤ 4 weeks)	NS	—	Wait time (> 4 vs. ≤ 4 weeks)	1.96 (1.10–3.50)	0.023
	—	—	—	Gender (Male vs. female)	2.38 (0.95–5.88)	0.063
	—	—	—	T category (T3-4 vs. T1-2)	4.34 (2.02–9.05)	< 0.001
	—	—	—	ACE-27 (> 1 vs. ≤ 1)	8.14 (2.39–27.68)	0.001
LRFS	Wait time (> 4 vs. ≤ 4 weeks)	NS	—	Wait time (> 4 vs. ≤ 4 weeks)	NS	—
	Age (> 45 vs. ≤ 45)	2.62 (0.70–9.90)	0.155	N category (N1-3 vs. N0)	2.56 (0.92–7.17)	0.073
	—	—	—	ACE-27 (> 1 vs. ≤ 1)	3.86 (0.93–15.98)	0.062

Abbreviations: ACE-27 = Adult Comorbidity Evaluation-27, CI = confidence interval, DMFS = distant metastasis-free survival, HR = hazard ratio, LRFS = locoregional recurrence-free survival, NACT = neoadjuvant chemotherapy, NS = not significant, OS = overall survival, PFS = progression-free survival.

a *P*-values were calculated using an adjusted Cox proportional hazards model.

### Prognostic value of the wait time for patients without NACT stratified according to early and advanced NPC stages

To test the individual hypothesis, we stratified patients without NACT according to the clinical stage into early (stage I + II) and advanced stage (stage III + IVa-b) groups; 104 and 184 pairs of NPC patients were respectively selected by PSM (Supplementary Table S2).

**Figure 3:**
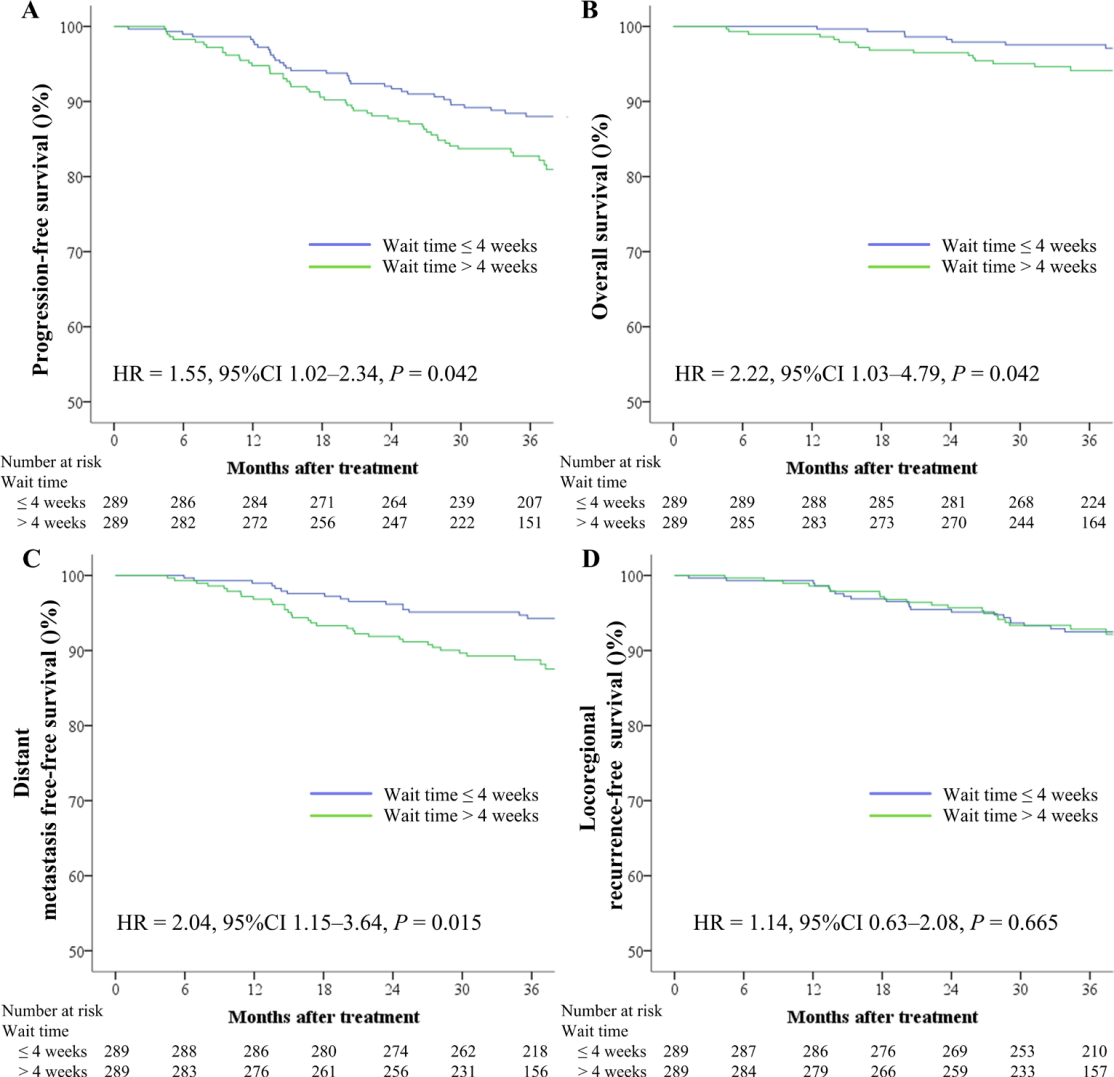
Kaplan–Meier survival curves based on the wait time for progression-free survival (**A**), overall survival (**B**), distant metastasis-free survival (**C**), and locoregional recurrence-free survival (**D**) rates for 184 pairs of patients with locoregionally advanced nasopharyngeal carcinoma who did not receive neoadjuvant chemotherapy. CI = confidence interval, HR = hazard ratio.

In the early stage group, the 3-year PFS (92.8% vs. 92.9%, respectively; *P* = 0.990), OS (97.1% vs. 99.0%, respectively; *P* = 0.316), DMFS (94.5% vs. 95.8%, respectively; *P* = 0.732), and LRFS (98.1% vs. 93.4%, respectively; *P* = 0.157) rates were comparable between patients with wait times of > 4 weeks and ≤ 4 weeks. In the advanced stage group, the 3-year PFS (78.9% vs. 86.1%, respectively; *P* = 0.040; Figure 2A) and DMFS (85.9% vs. 93.3%, respectively; *P* = 0.028; Figure 2C) were significantly lower for patients with a wait time of > 4 weeks than for those with a wait time of ≤ 4 weeks, while the 3-year OS rate exhibited a tendency to be lower in the former than in the latter (92.4% vs. 96.7%, respectively; *P* = 0.088; Figure 3B). There were no significant differences in the 3-year LRFS rate between patients with a wait time of > 4 weeks and those with a wait time of ≤ 4 weeks (89.8% vs. 92.0%; *P* = 0.177; Figure 3D). Multivariate analysis showed that a wait time of > 4 weeks was an independent unfavorable prognostic factor for PFS and DMFS in patients with advanced NPC without NACT (Table 4).

**Table 4:** Multivariate analysis of prognostic factors for patients without NACT stratified according to early and advanced stages of nasopharyngeal carcinoma

Endpoint	Patients with NACT (*n* = 104 pairs)			Patients without NACT (*n* = 184 pairs)		
	Variable	HR (95% CI)	*P* ^a^	Variable	HR (95% CI)	*P* ^a^
PFS	Wait time (> 4 vs. ≤ 4 weeks)	NS	—	Wait time (> 4 vs. ≤ 4 weeks)	1.66 (1.02–2.71)	0.043
	T category (T2 vs. T1)	1.95 (0.68–5.61)	0.218	N category (N1-3 vs. N0)	4.29 (1.05–17.50)	0.043
OS	Wait time (> 4 vs. ≤ 4 weeks)	NS	—	Wait time (> 4 vs. ≤ 4 weeks)	2.11 (0.89–5.00)	0.088
DMFS	Wait time (> 4 vs. ≤ 4 weeks)	NS	—	Wait time (> 4 vs. ≤ 4 weeks)	2.08 (1.08–3.99)	0.028
	ACE-27 (> 1 vs. ≤ 1)	6.01 (0.75–48.05)	0.091	Gender (Male vs. female)	3.03 (0.93–10.00)	0.067
LRFS	Wait time (> 4 vs. ≤ 4 weeks)	NS	—	Wait time (> 4 vs. ≤ 4 weeks)	NS	—
	ACE-27 (> 1 vs. ≤ 1)	7.48 (0.92–60.92)	0.060	N category (N1-3 vs. N0)	4.35 (0.60–31.82)	0.148

Abbreviations: ACE-27 = Adult Comorbidity Evaluation-27, CI = confidence interval, DMFS = distant metastasis-free survival, HR = hazard ratio, LRFS = locoregional recurrence-free survival, NACT = neoadjuvant chemotherapy, NS = not significant, OS = overall survival, PFS = progression-free survival.

a *P*-values were calculated using an adjusted Cox proportional hazards model.

## DISCUSSION

To the best of our knowledge, this is the first study to examine the association of the time between diagnosis and primary treatment, i.e., the wait time, with prognosis in a large population-based NPC cohort treated with IMRT. Using PSM and multivariate analyses, this study provides the fairest comparisons of matched patients and demonstrated higher 3-year PFS rates for NPC patients who received IMRT within 4 weeks of diagnosis than for those who received IMRT after 4 weeks. Further stratified analyses revealed that the negative effects of a prolonged wait time (> 4 weeks) occurred primarily in patients with advanced NPC without NACT. Finally, the wait time had no significant effects on the 3-year LRFS rate.

Despite the various growth rates for different tumors, a prolonged time between diagnosis and definitive primary treatment increases the likelihood of tumor growth and progression, which can facilitate both local invasion and distant metastasis, including deeper lymphovascular space involvement, and can result in a poor prognosis [16]. The lack of influence of the wait time on the 3-year LRFS rate can be attributed to the use of IMRT, which greatly improves locoregional control in NPC patients. However, a prolonged wait time increased distant failures for patients with advanced NPC without NACT. Although a causal relationship between the wait time and prognosis could not be established, our results suggest that a prolonged time between NPC diagnosis and radical RT as the primary treatment results in a poor prognosis by facilitating distant invasion, particularly in patients with advanced disease who receive no additional NACT. A recent meta-analysis indicated that additional NACT can effectively decrease the rate of distant metastasis [5]. Accordingly, the wait time did not influence the survival rates for patients with NACT, even though these patients exhibited a prolonged wait time. Besides, locoregionally advanced NPC progresses faster, and a delay in RT for patients with no additional NACT can result in a poor prognosis.

A clear understanding of the factors associated with a prolonged wait time can aid clinicians in providing better care. A recent study found that NPC patients with more comorbidities were associated with a prolonged wait time [6]. Comorbidities, quantified by the Adult Comorbidity Evaluation-27 (ACE-27), negatively influenced the quality of life, cost of treatment, and therapeutic decision-making for patients with head and neck cancers in previous studies [17, 18]. We also reported that comorbidities assessed by ACE-27 significantly worsened the prognosis of NPC patients [19]. The specific causes of a prolonged wait time in our study were unknown, and the presence of comorbid conditions may have played a major role. However, after adjusting for ACE-27 scores using PSM, the wait time was found to be an independent unfavorable prognostic factor. Furthermore, our institution is located in southern China, an endemic area with a large number of NPC patients that leads to hospital crowding and less availability of RT instruments. This may also have accounted for the prolonged wait time. Individual health care providers can decrease the wait time by optimizing the number and use of RT instruments. Moreover, future policies should aim at providing radical RT within a month of diagnosis to improve the survival rates for NPC patients, particularly those who do not receive additional NACT.

The major strength of our study is the use of PSM and multivariate analyses to investigate the influence of the wait time on NPC prognosis; this addressed the potential limitations of divergent confounders, treatment heterogeneity, and selection bias associated with the direct retrospective analysis of observational data [15]. With regard to the limitations, first, the presented data were derived from a single institution located in an endemic area with expertise in NPC. Second, there was no randomization; therefore, some imbalance is inevitable. However, only a retrospective design can evaluate the risks associated with a prolonged wait time, and we used PSM to minimize potential bias. Third, we enrolled NPC patients treated with IMRT in recent years, leading to the lack of long-term follow-up results. A shorter follow-up period produces fewer events, preventing data from reaching statistical significance. This may explain why the wait time did not influence the 3-year OS in our study. However, the 3-year PFS is an acceptable surrogate endpoint for NPC and can also predict the 5-year OS [20]. Nevertheless, further studies with longer follow-up durations are required to further clarify our findings. Finally, data such as specific socioeconomic status and patient preference were not available to us, and we could not identify the precise causes for a prolonged wait time. Pre-treatment Epstein-Barr virus deoxyribonucleic acid level may also affect the prognostic value of wait time. Future studies should also address these issues.

In conclusion, our results suggest that a prolonged time (> 4 weeks) between diagnosis and primary radical radiotherapy is a disadvantage for NPC patients, particularly those with advanced disease receiving no NACT. However, it is difficult to establish the optimal time for RT initiation after examination by a radiation oncologist. Given the present evidence, it is recommended that the wait time should not exceed 4 weeks for patients with locoregionally advanced NPC who do not receive NACT. It is necessary to optimize resources for decreasing this wait time, although additional studies are warranted to further clarify our findings.

## MATERIALS AND METHODS

### Ethics statement

The investigation has been conducted in accordance with ethical standards and according to the Declaration of Helsinki and according to national and international guidelines and has been approved by the ethics committee of Sun Yat-Sen University Cancer Center (SYSUCC). As this was a retrospective analysis of routine data, we were granted a waiver of written consent, and verbal consent was obtained from the patients.

### Patients

In total, 1811 patients with newly diagnosed, biopsy-proven, nonmetastatic NPC treated with IMRT at SYSUCC between October 2009 and February 2012 were retrospectively reviewed. Of these, 118 patients were excluded because of the lack of substantial information regarding the date of diagnosis or the date of RT initiation, while 21 were excluded because of insufficient information regarding comorbidities. The remaining 1672 patients were included in the current study. All patients underwent a comprehensive pretreatment evaluation, including a complete history, physical examination, hematology and biochemistry profiles, magnetic resonance imaging (MRI) of the neck and nasopharynx, chest radiography, abdominal sonography, and whole-body bone scanning using single-photon emission computed tomography (SPECT). Additional positron emission tomography/computed tomography (PET-CT) was performed for 485/1672 (29.0%) patients. All patients were restaged according to the seventh edition of the International Union against Cancer/American Joint Committee on Cancer (UICC/AJCC) staging system [21].

### Treatment

The nasopharyngeal and neck tumor volumes of all patients were treated using IMRT for the entire course. Target volumes were delineated slice-by-slice on treatment-planning CT scans using an individualized delineation protocol that complied with the International Commission on Radiation Units and Measurements reports 50 and 62. The prescribed doses were 66–72 Gy in 28–33 fractions to the planning target volume (PTV) of the primary gross tumor volume (GTVnx), 64–70 Gy to the PTV of the GTV of the involved lymph nodes (GTVnd), 60–63 Gy to the PTV of the high-risk clinical target volume (CTV1), and 54–56 Gy to the PTV of the low-risk clinical target volume (CTV2). All targets were simultaneously treated using the simultaneous integrated boost technique. All patients were treated with one fraction daily over 5 days per week; other details of the techniques used at our center have been reported previously [2].

During the study, the institutional guidelines recommended only IMRT for stage I disease and CCRT ± NACT/AC for stage II–IVB disease. NACT or AC included cisplatin with taxane, cisplatin with 5-fluorouracil, or triplet chemotherapy with cisplatin and taxane plus 5-fluorouracil every 3 weeks for two to three cycles. CCRT included cisplatin administered at weeks 1, 4, and 7 of RT or weekly. In total, 1141/1219 (93.6%) patients with stage III–IVB NPC received CCRT ± NACT/AC. Reasons for incompliance included refusal by individual patients or age or organ dysfunction suggestive of intolerance to treatment. When possible, salvage treatments (intracavitary brachytherapy, surgery, or chemotherapy) were provided for documented relapse or persistent disease.

### Definitions of variables

The wait time was defined as the number of days between the date of diagnosis (date of biopsy) and the date of definitive RT. A cutoff value of > 4 weeks was used to define a prolonged wait time [6]. Comorbidities were assessed using the ACE-27 [19], which grades specific conditions on the basis of organ system decompensation using a three-point scale: 0 = none, 1 = minimal, 2 = moderate, and 3 = severe.

### Follow-up and statistical analysis

The follow-up duration was measured from the first day of treatment to the day of last examination or death. All patients were examined every 3 months during the first 2 years, with follow-up examinations every 6 months for 3 years thereafter or until death. The primary endpoint was PFS, defined as the time from the first day of therapy to the date of disease progression or death from any cause. The secondary endpoints included OS, defined as the time from the first day of therapy to the date of the last follow-up or death from any cause, and DMFS and LRFS, defined as the time from the first day of therapy to the date of detection of distant metastasis and locoregional recurrence, respectively.

We selected patients with a wait time of > 4 weeks who were matched with those with a wait time of ≤ 4 weeks using PSM, a method for creating similar case (wait time > 4 weeks) and control (wait time ≤ 4 weeks) sets from an existing dataset of the presumed covariates to minimize possible bias in a retrospective analysis [22]. Propensity scores were computed by logistic regression for each patient using the following covariates: age, sex, chemotherapy regimen, T-stage, N-stage, clinical stage, and ACE-27 score. Patients with a wait time of > 4 weeks and ≤ 4 weeks were then matched without replacement at the ratio of 1:1 for those scores, rather than the individual covariates. PSM was also utilized to identify matched patients in subsequent stratified analyses.

SPSS (version 19.0; SPSS Inc, Chicago, Ill), and STATA version 12.0 (Stata Corporation, College Station, TX) were used for all statistical analyses. Covariate balance between matched groups was examined by chi-square tests or Fisher's exact tests when indicated. Kaplan–Meier curves were used to estimate the actuarial rates and log-rank tests were used for comparisons [23]. Multivariate analyses using Cox proportional hazards model were used to test for independent significance by backward elimination of insignificant explanatory variables [24]. The following parameters were included in the model as covariates: age (> 45 years vs. ≤ 45 years), sex (male vs. female), chemotherapy (with vs. without), T-stage (T3–4 vs. T1–2), N-stage (N1–3 vs. N0), and ACE-27 score (>1 vs. ≤1). Two-tailed *P*-values of <0.05 were considered statistically significant.

## SUPPLEMENTARY FIGURES AND TABLES


