# Supplementary material for: Prognostic value of wait time in nasopharyngeal carcinoma treated with intensity modulated radiotherapy: a propensitymatched analysis
Source: Oncotarget. 2016 Feb 29;7(12):14973–82. doi: 10.18632/oncotarget.7789 (PMC4924766; doi:10.18632/oncotarget.7789)
Supplement: Supplementary file 1 [file oncotarget-07-14973-s001.pdf]

Prognostic value of wait time in nasopharyngeal carcinoma treated with intensity modulated radiotherapy: a propensity-matched analysis

Supplementary Materials

Supplementary Table S1: Baseline characteristics of patients with nasopharyngeal carcinoma with and without NACT

Characteristic	Patients with NACT			Patients without NACT		
	Wait time ≤ 4 weeks (*n* = 40) No. (%)	Wait time > 4 weeks (*n* = 40) No. (%)	*P* [a]	Wait time ≤ 4 weeks (*n* = 289) No. (%)	Wait time > 4 weeks (*n* = 289) No. (%)	*P* [a]
Age			1.000			0.933
≤ 45	21 (52.5)	21 (52.5)		160 (55.4)	161 (55.7)	
> 45	19 (47.5)	19 (47.5)		128 (44.6)	128 (44.3)	
Sex			0.799			0.843
Male	29 (72.5)	30 (75.0)		222 (76.8)	224 (77.5)	
Female	11 (27.5)	10 (25.0)		67 (23.2)	65 (22.5)	
WHO pathology			1.000			0.499
Type I	0 (0)	1 (2.5)		2 (0.7)	0 (0)	
Type II/III	40 (100.0)	39 (97.5)		287 (99.3)	289 (100.0)	
T category			0.983			0.608
T1	7 (17.5)	7 (17.5)		73 (25.3)	72 (24.9)	
T2	6 (15.0)	5 (12.5)		51 (17.6)	52 (18.0)	
T3	21 (52.5)	21 (52.5)		135 (46.7)	125 (43.3)	
T4	6 (15.0)	7 (17.5)		30 (10.4)	40 (13.8)	
N category			1.000			0.992
N0	7 (17.5)	7 (17.5)		58 (20.1)	58 (20.1)	
N1	24 (60.0)	25 (62.5)		167 (57.8)	164 (56.7)	
N2	5 (12.5)	5 (12.5)		44 (15.2)	46 (15.9)	
N3	4 (10.0)	3 (7.5)		20 (6.9)	20 (7.3)	
Clinical stage			1.000			0.759
I	1 (2.5)	1 (2.5)		25 (8.7)	24 (8.3)	
II	8 (20.0)	8 (20.0)		81 (28.0)	79 (27.3)	
III	21 (52.5)	21 (52.5)		135 (46.7)	128 (44.3)	

IV	10 (25.0)	10 (25.0)		48 (16.6)	58 (20.1)	
Chemotherapy			—			0.390
No	0 (0)	0 (0)		69 (23.9)	78 (27.0)	
Yes	40 (100.0)	40 (100.0)		220 (76.1)	211 (73.0)	
ACE-27			1.000			0.286
≤ 1	39 (97.5)	39 (97.5)		288 (99.3)	282 (97.9)	
> 1	1 (2.5)	1 (2.5)		2 (0.7)	6 (2.1)	

Abbreviations: ACE-27 = Adult Comorbidity Evaluation-27; NACT = neoadjuvant chemotherapy; WHO = World Health Organization.
[a]*P*-values were calculated using chi-square tests or Fisher's exact test when indicated.

Supplementary Table S2: Baseline characteristics of patients without NACT stratified according to early and advanced stages of nasopharyngeal carcinoma

Characteristic	Early stage (I + II)			Advanced stage (III + IVa–b)		
	Wait time ≤ 4 weeks (*n* = 104) No. (%)	Wait time > 4 weeks (*n* = 104) No. (%)	*P* [a]	Wait time ≤ 4 weeks (*n* = 184) No. (%)	Wait time > 4 weeks (*n* = 184) No. (%)	*P* [a]
Age			1.000			0.751
≤ 45	54 (51.9)	54 (51.9)		109 (59.2)	106 (57.6)	
> 45	50 (48.1)	50 (48.1)		75 (40.8)	78 (42.4)	
Sex			0.760			0.895
Male	75 (72.1)	73 (70.2)		148 (80.4)	149 (81.0)	
Female	29 (27.9)	31 (29.8)		36 (19.6)	35 (19.0)	
WHO pathology			0.316			1.000
Type I	1 (1.0)	0 (0)		1 (0)	0 (0)	
Type II/III	103 (99.0)	104 (100.0)		183 (99.5)	184 (100.0)	
T category			0.672			0.370
T1	60 (57.7)	63 (60.6)		12 (6.5)	8 (4.3)	
T2	44 (42.3)	41 (39.4)		6 (3.3)	11 (6.0)	
T3	—	—		130 (70.7)	122 (66.3)	
T4	—	—		36 (19.6)	43 (23.4)	
N category			0.886			0.997
N0	38 (36.5)	39 (37.5)		21 (11.4)	20 (10.9)	
N1	66 (63.5)	65 (62.5)		99 (53.8)	101 (54.9)	
N2	—	—		43 (23.4)	42 (22.8)	
N3	—	—		21 (11.4)	21 (11.4)	
Clinical stage			0.868			0.574
I	23 (22.1)	24 (23.1)		—	—	

II	81 (77.9)	80 (76.9)		—	—	
III	—	—		129 (70.1)	124 (67.4)	
IV	—	—		55 (29.9)	60 (32.6)	
Chemotherapy			0.579			0.661
No	54 (51.9)	50 (48.1)		26 (14.1)	29 (15.8)	
Yes	50 (48.1)	54 (51.9)		158 (85.9)	155 (84.2)	
ACE-27			1.000			0.724
≤ 1	102 (98.1)	102 (98.1)		181 (98.4)	180 (97.8)	
> 1	2 (1.9)	2 (1.9)		3 (1.6)	4 (2.2)	

Abbreviations: ACE-27 = Adult Comorbidity Evaluation-27; NACT = neoadjuvant chemotherapy; WHO = World Health Organization.

[a]*P*-values were calculated using chi-square tests or Fisher's exact tests when indicated.
